# T cell-intrinsic IL-1R signaling licenses effector cytokine production by memory CD4 T cells

**DOI:** 10.1038/s41467-018-05489-7

**Published:** 2018-08-09

**Authors:** Aakanksha Jain, Ran Song, Edward K. Wakeland, Chandrashekhar Pasare

**Affiliations:** 1 Department of Immunology, University of Texas Southwestern Medical Center, Dallas, TX 75390 USA; 2 Division of Immunobiology, Cincinnati Children’s Hospital Medical Center, Cincinnati, OH 45229 USA

## Abstract

Innate cytokines are critical drivers of priming and differentiation of naive CD4 T cells, but their functions in memory T cell response are largely undefined. Here we show that IL-1 acts as a licensing signal to permit effector cytokine production by pre-committed Th1 (IFN-γ), Th2 (IL-13, IL-4, and IL-5) and Th17 (IL-17A, IL-17F, and IL-22) lineage cells. This licensing function of IL-1 is conserved across effector CD4 T cells generated by diverse immunological insults. IL-1R signaling stabilizes cytokine transcripts to enable productive and rapid effector functions. We also demonstrate that successful lineage commitment does not translate into productive effector functions in the absence of IL-1R signaling. Acute abrogation of IL-1R signaling in vivo results in reduced IL-17A production by intestinal Th17 cells. These results extend the role of innate cytokines beyond CD4 T cell priming and establish IL-1 as a licensing signal for memory CD4 T cell function.

## Abstract (web summary)

CD4 T cell polarizations and functions are regulated by cytokines from innate cells. Here the authors show that IL-1 deficiency does not impair the differentiation of Th1, Th2 and Th17, but IL-1 signaling is required for maintaining the expressions of their respective key cytokines to ‘license’ the functions of these T cell subsets.

## Introduction

Pathogen recognition by dendritic cells (DCs) via activation of pattern recognition receptors (PRRs) results in the upregulation of MHC and co-stimulatory molecules and the secretion of pro-inflammatory cytokines^1,2^. The MHC-peptide complex ensures cognate T cell activation while upregulation of co-stimulatory markers reflect the non-self nature of the antigen^3^. Although TCR engagement and co-stimulation can lead to activation and proliferation of CD4 T cells, innate cytokines are required for differentiation of naive T cells into differentially programmed protective subsets tailored to eliminate specific microbial challenges^4^.

Following pathogen clearance, antigen-specific T cells survive as memory T cells or effector T cells that either recirculate or reside permanently in the tissues^5^. In contrast to the three signal requirement for naive T cell priming^6^, it has long been presumed that MHC-TCR interactions alone are sufficient for memory CD4 T cell reactivation and effector function^7,8^. More recent work, however, has shown that co-stimulation via CD80/86 is also critical for reactivation of memory CD4 T cells^9–11^. While dependence on only one (or two) signal(s) might allow for rapid reactivation of previously primed T cells, insufficient stringency can be inherently dangerous to the host physiology^12^. Using yeast-displayed MHC-peptide libraries, it was found that a single TCR could bind to several related peptides^13^; later it was shown that pathogenic peptides could cross-react with self-reactive TCRs^14^. Thus, pathogen-specific memory T cells are prone to aberrant reactivation by self-antigens. The risk of such undesirable activation is even higher at the barrier surfaces where tissue resident memory T cells are exposed to innocuous tissue restricted self-peptides^15,16^. Lack of qualitative information about the origin of the antigen during effector or memory T cell reactivation could thus lead to systemic or local auto-immune and auto-inflammatory responses^17^. This inspired us to hypothesize that the innate immune system provides additional cues for memory T cell reactivation beyond antigen presentation and co-stimulation. We propose that cues from the innate immune system regulate T cell responses at stages past differentiation by providing qualitatively distinct signals during naive T cell priming versus memory T cell reactivation.

Innate cytokines can be broadly categorized based on their dependence on STAT or MyD88-mediated signaling. Priming cytokines such as IL-6, IL-12 and IL-4 signal via the activation of STAT molecules, which induce or stabilize the expression of lineage specific transcription factors^6^. On the other hand, the IL-1 family of cytokines, including IL-1α, IL-1β, IL-18 and IL-33, engage MyD88-dependent signaling to enhance T cell responses that are primarily orchestrated by priming cytokines^6,18^. Before even the biology of its production and signaling was completely understood, IL-1 was predicted and shown to be a critical signal for T helper cell growth^19^. While at the time CD4 T cell subsets were yet to be discovered, IL-1 was thought to be a signal secondary to TCR activation^19,20^. In agreement with this hypothesis, it was subsequently shown that T cell-intrinsic MyD88 is necessary to block Treg suppression of effector CD4 T cell immunity^21^. Over the years various other unique roles of IL-1 and its related cytokines have been described. More specifically, IL1β was shown to enhance Th17 differentiation in vitro^22^. IL-1R signaling promotes pathogenicity of autoimmune Th17 cells by synergizing with IL-6 and IL-23^23^. More importantly, IL-1R signaling was required for Th17 immunity irrespective of the tissue microenvironment^18^. This finding underscores IL-1 as a central signal rather than a secondary cue for Th17 mediated immunity. The role of IL-1 family of cytokines extends to other CD4 T cell lineages as well. IL-18 aids Th1 priming by upregulating T-bet expression^24^. IL-18 in conjunction with IL-12 can also induce antigen independent memory CD8 and CD4 T cell effector function^25,26^. IL-33 has an established role in promoting Th2 immunity^27^ and more recently has been shown to enhance tissue resident Treg function^28^. In summary, diverse yet unique functions of the IL-1 family of cytokines have been reported in all effector CD4 T cell lineages, giving rise to the following questions. First, is there a common principle that underlines the dependence of all CD4 T cell lineages on the IL-1 family of cytokines for protective immunity? Second, why did T cells adopt a MyD88-dependent pathway, which is analogous to Toll-like receptor (TLR) signaling, during the course of evolution of the adaptive immune system?

It is crucial to note that in the aforementioned studies, IL-1 or its related cytokines were either exogenously provided to an ongoing T cell response, which does not reveal the necessity of these cytokines for CD4 T cell function or IL-1R signaling components were genetically ablated in animal models, which makes it challenging to differentiate between the function of IL-1 related cytokines during T cell priming versus reactivation. Here, we explored the role of the IL-1 family of cytokines in regulating effector function of memory T cells by specifically ablating signaling downstream of the IL-1 family of receptors during reactivation of already primed effector or memory T cells. This methodology enables exclusion of the previously confounding factors.

Our data demonstrate that T cell-intrinsic signaling downstream of IL-1 and IL-18, act as “licensing” signal for cytokine production by memory CD4 T cells. More importantly, the necessity for this licensing cytokine cue is conserved across all effector CD4 T cell lineages. We find that while *Il1r*^*−/−*^ CD4 T cells can differentiate into Th1, Th2 and Th17 lineages at steady state in vivo, they are unable to secrete effector cytokines following TCR ligation, underscoring the requirement for IL-R signaling during reactivation of committed CD4 T cells. Furthermore, IL-1R signaling in T cells provides post-transcriptional stability to otherwise unstable T cell cytokine transcripts, analogous to TLR mediated stabilization of innate cytokine transcripts in myeloid cells. Together, our data show that T cell-intrinsic IL-1R signaling is a critical cue rather than an accessory signal for productive T cell effector function and provide a previously unrecognized evolutionary basis for adaptation of MyD88 signaling pathway in T cells.

## Results

### DCs provide key signals for CD4 T cell effector function

**Fig. 1:**
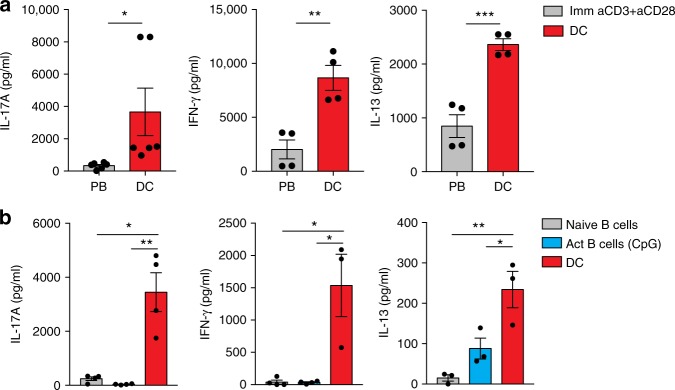
Dendritic cells provide key signals necessary for optimal functioning of effector CD4 T cells. **a** CD44^hi^ CD62L^lo^ CD4 T cells (T_em_) were stimulated with soluble αCD3 (30 ng ml^−1^) in the presence of either live splenic CD11C + DCs or αCD3 (0.5 μg ml^−1^) and αCD28 (0.5 μg ml^−1^) antibodies immobilized on a plate. **b** CD44^hi^ CD62L^lo^ CD4 T cells (T_em_) were stimulated with soluble αCD3 (30 ng ml^−1^) in the presence of either live DCs, naive and activated (CpG-stimulated) CD19 + B cells. Cytokine levels in the culture supernatants were determined after 48 h of activation using paired-antibody ELISAs. Error bars indicate SEM; paired *t* test

It has long been understood that engagement of TCR and co-stimulatory molecules on antigen-experienced CD4 T cells (effector, effector memory and tissue resident memory cells) is sufficient to drive rapid production of effector cytokines^8,10^. This is based on the conceptual framework that once primed, CD4 T cells need not depend on additional innate immune signals as this would impose a degree of redundancy and might delay the effector response. We formally tested this concept by either strictly providing TCR and co-stimulatory signals (using plate-bound antibodies) to circulating effector memory CD4 T cells (CD44^hi^CD62L^lo^, here on referred to as memory CD4 T cells) or by activating these cells in the presence of live DCs. Consistent with the existing paradigm, we found that engagement of the TCR and the co-stimulatory molecule, CD28, was sufficient for memory CD4 T cells to produce canonical Th1 (IFNγ), Th2 (IL-13) and Th17 (IL-17A) cytokines (Fig. 1a). However, when compared to the quantities of effector cytokines produced by memory CD4 T cells co-cultured with live DCs, the effector cytokines induced by plate-bound antibodies were significantly lower (Fig. 1a). This is remarkable since much higher concentrations of antibodies were used for plate bound stimulation compared to soluble αCD3 used to mediate DC-T cell interactions. We further asked if B cells, naive or previously activated by TLR ligands, would provide adequate signals for optimal functioning of these memory CD4 T cells. Surprisingly, we found that neither naive nor activated B cells could induce optimal cytokine production comparable to those induced by live DCs (Fig. 1b). Collectively, this set of experiments demonstrates that TCR and co-stimulatory signals are not sufficient to trigger optimal effector cytokine production by Th1, Th2 and Th17 cells and that additional signals, potentially resulting from a complex cross-talk between memory CD4 T cells and live DCs, could be critical for T cell effector function.

### Effector CD4 T cells express receptors for IL-1 family

**Fig. 2:**
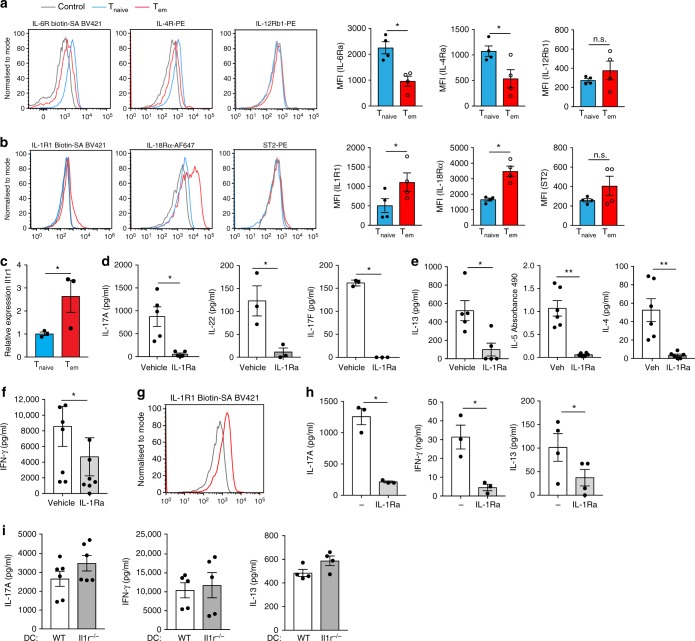
Effector CD4 T Cells constitutively express IL-1R and require IL-1R signaling for cytokine production. **a**, **b** Splenic CD4 T cells were gated on naive (T_naive_) and memory (T_em_) T cell markers and stained for **a** IL-6Ra, IL-4Ra and IL-12Rb1 **b** IL-1R1, IL-18Rα and IL-33R (ST2). **c** Relative expression of *Il1r1* transcripts in T_naive_ and T_em_ cells. Data is normalized to 18s. **d**-**f** T_em_ cells were stimulated with splenic DCs and soluble αCD3 (30 ng ml^−1^) in the presence or absence of IL-1R antagonist (IL-1Ra) for 48 h **d** IL-17A, IL-17F, IL-22, **e** IL-13, IL-4, IL-5, and **f** IFNγ were measured. **g** CD4 T cells from SI-LP were stained for IL-1R1. **h** CD4 T cells from SI-LP were stimulated using CD11C + DCs and soluble αCD3 (30 ng ml^−1^) in the presence or absence of IL-1Ra for 48 h followed by cytokine ELISA. **i** IL-17A, IL-13 and IFNγ levels in supernatants obtained from T_em_ cell co-cultures with WT or *Il1r*^*−/−*^CD11c + splenic DCs in the presence of αCD3 (30 ng ml^−1^) after 48 h of stimulation. Control in receptor staining refers to fluorescence minus one control. IL-17A, IL-13 and IFNγ in the culture supernatants were measured using paired-antibody ELISA. Error bars indicate SEM; paired *t* test, **c**–**f**, **h**, **i** 4–8 mice were pooled per experiment

T cell receptor activation and co-stimulatory signaling activate and induce clonal expansion of CD4 T cells. Naive CD4 T cells, in addition, depend on cytokine signals for differentiation into protective subsets. The role of innate cytokines in CD4 T cell responses can be postulated in part by analyzing the expression of their receptors on CD4 T cells. The receptors for priming cytokines (especially IL-6 and IL-4) were expressed constitutively on naive CD4 T cells but were downregulated on effector memory CD4 T cells (Fig. 2a and Supplementary Fig. 1a). IL-12R was not constitutively expressed on naive CD4 T cells but was induced following TCR stimulation and then rapidly downregulated (Fig. 2a and Supplementary Fig. 2). However, when we examined the expression of receptors for the IL-1 family of cytokines, IL-1R, IL-18R and IL-33R, we found that IL-1R and IL-18R were constitutively expressed on effector memory CD4 T cells in the secondary lymphoid organs (Fig. 2b and Supplementary Fig. 1a). Interestingly, circulating effector memory CD4 T cells did not show significant expression of ST2, the receptor for IL-33 (Fig. 2b and Supplementary Fig. 1a). It is well known that in vivo generated Th2 cells can respond to IL-33^29^ and perhaps IL-33R could be regulated by different mechanisms than those that regulate IL-1 and IL-18 receptors. In agreement with IL-1R staining, transcriptional expression of IL-1R was also higher on effector memory CD4 T cells relative to their naive counterparts (Fig. 2c). Collectively, these data led us to consider the possibility that signals from the IL-1 family of cytokines, particularly IL-1, could be critical for regulating effector function of previously differentiated CD4 T cells.

### IL-1R signaling is critical for CD4 T cell effector functions

To test the importance of constitutive expression of IL-1R on memory CD4 T cells, we specifically blocked IL-1R signaling only during the reactivation phase of WT memory CD4 T cells using IL-1R antagonist (IL-1Ra), a potent biological competitive inhibitor of both IL-1α and IL-1β. This approach ensures optimal in vivo T cell differentiation under IL-1 sufficient conditions and uncouples effector function from priming, which is not possible when using genetic deletion of the receptor or downstream signaling molecules. Abrogation of IL-1R signaling during memory T cell reactivation led to greatly diminished production of IL-17A, IL-17F and IL-22 (Fig. 2d). We then decided to test if the requirement of IL-1R signaling for effector function also extends to other CD4 T cell lineages. Indeed, we found significant deficit in IL-13, IL-4 and IL-5 as well as IFNγ production when we abrogated IL-1R signaling (Fig. 2e, f). IL-18 has been implicated in driving IFNγ production by CD8 T cells^30,31^; however, blocking IL-18R signaling did not affect IFNγ production by memory CD4 T cells (Supplementary Fig. 3a). Likewise, IL-33 neutralization did not significantly affect IL-13 production by memory CD4 T cells (Supplementary Fig. 3b). Requirement of IL-1R signaling appears to be specific for effector cytokines since IL-2 production was normal (Supplementary Fig. 3c). Acute blockade of IL-1R signaling during the reactivation phase did not affect CD25, CD69, ICOS and CD44 expression, suggesting that effector cytokine production was compromised despite normal T cell activation (Supplementary Fig. 3d). Like circulating memory T cells, small intestine lamina propria (SI-LP) resident CD69 + CD4 T cells also displayed constitutive expression of IL-1R (Fig. 2g, Supplementary Fig. 1b and Supplementary Fig 4). Consistently, blocking IL-1R inhibited Th1, Th2 and Th17 cytokine production by gut CD4 T cells during reactivation by DCs (Fig. 2h). The role of DC-intrinsic IL-1R signaling was excluded since we observed no defect in effector cytokine production when CD4 T cells were reactivated with *Il1r*^*−/−*^ DCs (Fig. 2i). These data indicate the existence of a common theme of innate regulation of T cell effector function conserved across all CD4 T cell lineages and provide evidence for an indispensable role for IL-1R signaling for effector function (as measured by their signature cytokine production) during the reactivation phase of pre-committed CD4 T cells.

### IL-1β licenses cytokine production by memory CD4 T cells

**Fig. 3:**
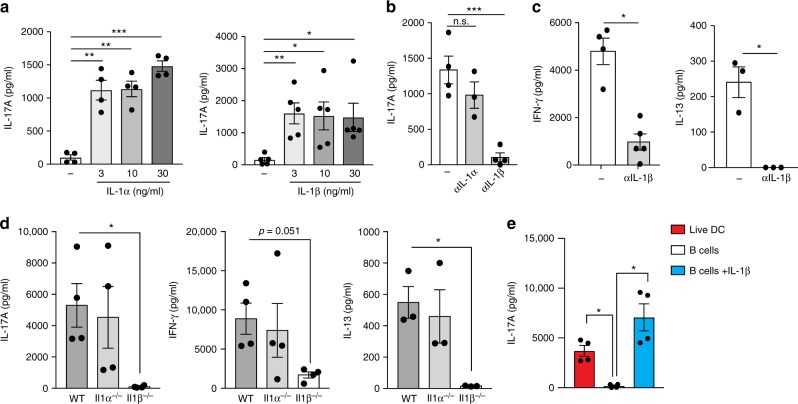
IL-1β licenses IL-17A, IFNγ and IL-13 production by circulating effector CD4 T cells. **a** CD44^hi^ CD62L^lo^ T_em_ cells were stimulated with immobilized αCD3 (0.5 μg ml^−1^) and αCD28 (0.5 μg ml^−1^) in the presence of indicated concentrations of recombinant IL-1α (left) and IL-1β (right). **b**, **c** T_em_ cells were co-cultured with splenic DCs and soluble αCD3 (30 ng ml^−1^) in the presence or absence of neutralizing antibodies against IL-1α or IL-1β (10 μg ml^−1^). **d** T_em_ cells from WT mice were stimulated with soluble αCD3 (30 ng ml^−1^) in the presence of splenic DCs from indicated genotypes. **e** T_em_ cells were stimulated with αCD3 (30 ng ml^−1^) using either splenic DCs or naive B cells as APCs in the presence or absence of recombinant IL-1β (10 ng ml^−1^). Cytokine concentrations in the culture supernatants were determined using paired-antibody ELISA. Error bars indicate SEM; paired *t* test

So far, we have used IL-1Ra to block IL-1R mediated signaling. Although both IL-1α and IL-1β utilize IL-1R for downstream signal transduction, these cytokines differ in how they are made and might in fact have different biological roles in regulating adaptive immunity^32^. IL-17A production by memory CD4 T cells activated via plate bound αCD3 and αCD28 was enhanced by addition of either IL-1α or IL-1β (Fig. 3a). However, the minimal cytokine production by memory CD4 T cells induced by TCR and co-stimulation alone was found to be independent of IL-1R signaling (Supplementary Fig. 5a). In order to establish which one of these cytokines is necessary for IL-17A production especially when CD4 T cells are interacting with DCs, we specifically neutralized IL-1α or IL-1β during CD4 T cell reactivation by live DCs. We found that IL-1β but not IL-1α was required for optimal cytokine production by Th17 cells (Fig. 3b). Furthermore, IL-13 and IFNγ production was also significantly compromised upon specific neutralization of IL-1β (Fig. 3c). Consistently, *Il11*α^*−/−*^ DCs but not *Il11*β^*−/−*^ DCs were able to induce optimal IL-17A, IL-13 and IFNγ production by CD4 T cells (Fig. 3d). Moreover, exogenous IL-1β restored the ability of B cells to induce IL-17 production by memory T cells to the levels induced by live DCs (Fig. 3e). Notably, unlike cytokine production by memory CD8 T cells, which can be induced by IL-12 and IL-18 in absence of TCR ligation^26^, IL-1β driven cytokine production by CD4 T cells was strictly dependent on concurrent TCR activation and co-stimulation (Supplementary Fig. 5b). In fact, blocking CD28 interaction with co-stimulatory molecules completely abrogated the ability of memory T cells to produce cytokines suggesting that co-stimulation continues to be a hierarchically superior signal (Supplementary Fig. 5c). Collectively, our data has revealed that IL-1β acts as a “licensing cytokine” for effector function of memory CD4 T cells of all lineages thus providing an additional layer of regulation of adaptive immune responses.

### IL-1R signaling enables antigen-specific CD4 T cell function

**Fig. 4:**
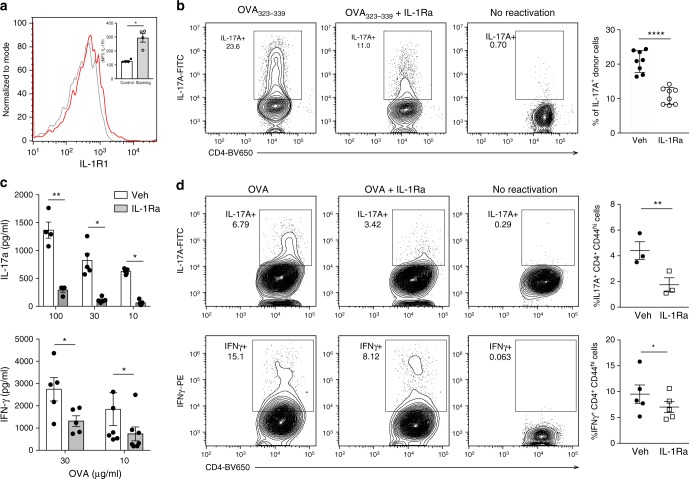
IL-1R signaling is necessary for effector cytokine production of newly primed CD4 T cells in the draining lymph nodes following subcutaneous immunization. **a**, **b** 5 × 10^5^ naive CD45.2 OT-II T cells were transferred into CD45.1 WT mice intravenously. The next day, recipient mice were immunized with OVA + LPS emulsified in IFA. **a** 7 days post-immunization, IL-1R expression on CD45.2 donor T cells from draining lymph nodes was examined. “Control” refers to fluorescence minus one control. **b** OT-II T cells from draining lymph nodes were reactivated with OVA_323-339_ for 12 h. Brefeldin A was added in the last 6 h of stimulation. Intracellular staining for IL-17A was performed following fixation and permeabilization of stimulated cells. Data are gated on live, CD4 + , CD45.2 + T cells. **c** CD4 + T cells from draining lymph nodes of mice immunized with LPS + OVA + IFA were reactivated using WT splenic DCs as antigen presenting cells in the presence of titrating doses of OVA for 48 h. IL-17A (top) and IFNγ (bottom) production was measured using paired antibody ELISA. **d** Total cells from draining LN of mice immunized with LPS + OVA + IFA were cultured with OVA (100 μg ml^−1^) for 18 h and brefeldin A was added during last 6 h of culture. Intracellular staining for IL-17A and IFNγ was performed following fixation and permeabilization of stimulated cells. Live CD4 + CD44^hi^ cells were gated and analyzed for intracellular expression of IL-17A or IFNγ. Unstimulated control cells serve as baselines for constitutive cytokine production by CD4 T cells. Error bars indicate SEM; paired *t* test

We, and others have previously shown that priming environments can have significant impact on the quality of CD4 T cells that are generated^18,33^. Moreover, the nature of the immune challenge can dictate innate cytokine requirements for CD4 T cell function^34^. Naturally arising memory CD4 T cells used so far in this study are most likely specific to commensals present at steady state^35^. Requirements for the generation and function of acutely activated T cells could be different than the ones primed under homeostasis^36,37^. Therefore, we decided to test if antigen-specific effector CD4 T cells generated following various routes of immune challenge also depend on IL-1R signaling for cytokine production, during secondary challenge. To this end, we transferred OT-II T cells into a WT host and immunized the recipient mice with ovalbumin (OVA) mixed with lipopolysaccharide (LPS) and incomplete Freund’s adjuvant for in vivo priming of the donor OT-II T cells. We observed that following immunization, there was constitutive IL-1R expression on primed and clonally expanded OT-II T cells (Fig. 4a and Supplementary Fig. 1c). Peptide-specific cytokine production by these OT-II T cells upon secondary stimulation with OVA peptide_323-339_ was dependent on IL-1R signaling (Fig. 4b and Supplementary Fig. 1c). We also generated polyclonal OVA-specific CD4 T cells following subcutaneous immunization. WT CD4 T cells isolated from the draining lymph nodes of the immunized mice were reactivated by live DCs in the presence of titrating doses of OVA. IFNγ and IL-17A production was heavily compromised upon IL-1R blockade during reactivation (Fig. 4c). Addition of exogenous IL-1β has been shown to promote CD4 T cell proliferation during priming^38^. However, we found no defect in the frequency of activated CD4 T cells as well as antigen-specific proliferation in the presence of IL-1Ra (Supplementary Fig. 6a, b). In line with secreted cytokines, the frequency of IL-17A + and IFNγ+ T cells in response to OVA re-stimulation was also significantly reduced when IL-1R signaling was abrogated (Fig. 4d and Supplementary Fig. 1d).

**Fig. 5:**
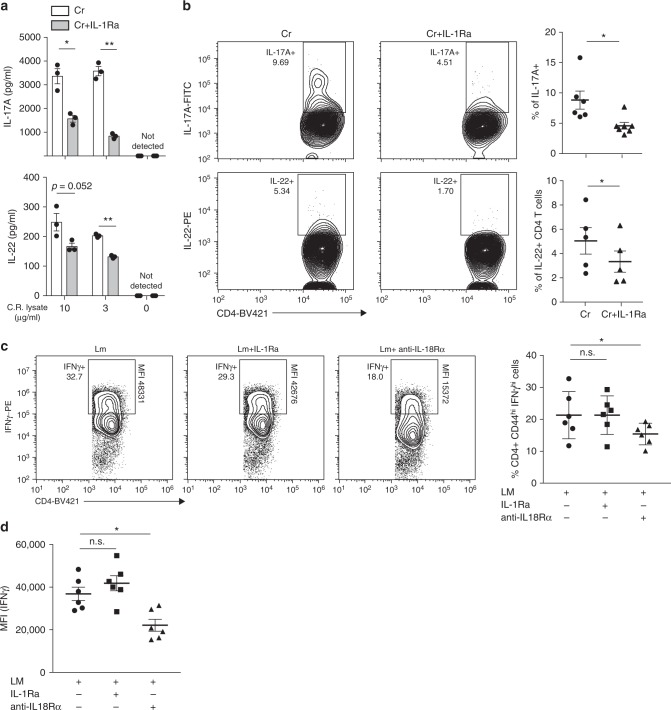
Antigen specific effector function of CD4 T Cells primed following oral and systemic infections is dependent on IL-1R and IL-18R signaling. **a** CD4 + T cells from mesenteric lymph nodes of *C. rodentium* infected mice were re-stimulated using WT splenic DCs as antigen presenting cells in the presence of titrating concentrations of *C. rodentium* lysate for 48 h. IL-17A and IL-22 production was measured using paired antibody ELISA. **b** Intracellular staining for IL-17A (top) and IL-22 (bottom) following pathogen-specific re-stimulation in the presence of brefeldin A. Live CD4 + CD44^hi^ cells were gated to examine intracellular expression of IL-17A and IL-22. **c** CD4 T cells from spleens of *L. monocytogenes* infected mice were cultured in the presence of live DCs and *L. monocytogenes* lysate. Intracellular staining for IFNγ following *L. monocytogenes* specific re-stimulation in the presence of brefeldin A. Live CD4 + CD44^hi^ cells were gated to examine intracellular expression of IFNγ under various conditions. **d** MFI quantification of IFNγ on CD4 + CD44hi cells following *L. monocytogenes-*specific re-stimulation under various conditions. Unstimulated control cells serve as baselines for constitutive cytokine production by CD4 T cells. Error bars indicate SEM; unpaired *t* test

Next, we challenged mice with *C. rodentium* using an intra-gastric route of infection. *C. rodentium* infection predominantly leads to Th17 and Th22 cell differentiation^39^. *C. rodentium* specific WT Th17 cells generated following intra-gastric infection also required IL-1R signaling for effector function as measured by IL-17A and IL-22 production following reactivation with *C. rodentium* lysate (Fig. 5a). The proportion of IL-17A+ as well as IL-22 + CD4 T cells was also significantly compromised in the presence of IL-1Ra (Fig. 5b and Supplementary Fig. 1d). No cytokine production was detected without antigen, discounting the possibility of any constitutive T cell response (Supplementary Fig. 6c). Lack of IL-17A production in T cells challenged with unrelated pathogen lysate further demonstrates the specificity of the effector T cell response in this assay (Supplementary Fig. 6c). Since oral and systemic infections have been shown to have different cytokine requirements for priming^18^, we wanted to test the requirement for IL-1R signaling in systemic infections. We infected WT mice with *L. monocytogenes* intra-peritoneally, which results in Th1-skewed responses^33,40^. CD4 T cells were isolated from the spleens of infected mice and re-stimulated in vitro with *L. monocytogenes* lysate for antigen-specific CD4 T cell reactivation. Interestingly, IFNγ production by *L. monocytogenes*-specific CD4 T cells was not affected by the absence of IL-1R signaling (Fig. 5c, d and Supplementary Fig. 1d). Since there is evidence for IL-18R signaling regulating production of IFNγ by both memory CD8 T cells and NK cells^18,26^, we tested whether *L. monocytogenes*-specific IFNγ production by effector CD4 T cells was dependent on IL-18R signaling. We found that ablation of IL-18R signaling significantly compromised IFNγ production (Fig. 5c, d and Supplementary Fig. 1d). Notably, the mean fluorescent intensity (MFI) of IFNγ was also reduced upon blocking IL-18R, suggesting the necessity of its signaling at the level of every cell (Fig. 5d). The activation of CD4 T cells upon pathogen-specific reactivation was normal in the absence of IL-1R or IL-18R signaling as determined by the frequency of activated CD4 T cells (Supplementary Fig. 6d, e). Collectively, these data demonstrate a critical role for IL-1R and IL-18R signaling in regulating antigen-specific effector function of CD4 T cells. In addition, these data also highlight how the nature of the pathogen and route of infection can dictate dependence of Th1 lineage cells on IL-1 or IL-18 signals.

### IL-1R signaling stabilizes T cell cytokine transcripts

**Fig. 6:**
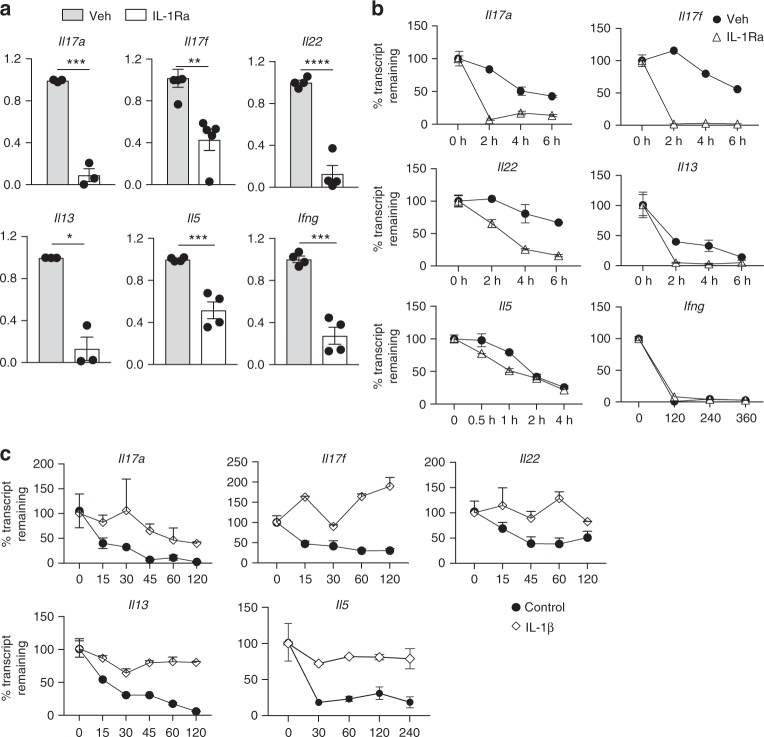
IL-1R signaling stabilizes Th17 and Th2 cytokine transcripts. **a** CD44^hi^ CD62L^lo^ T_em_ cells were stimulated by splenic DCs and soluble αCD3 (30 ng ml^−1^) in the presence or absence of IL-1Ra for 48 h. Respective cytokine transcripts were quantified in DC-T cell co-culture cell lysate. All data is normalized to 18 s and untreated control. **b** CD4 T cells were stimulated with CD11c + DCs and soluble αCD3. **c** CD4 T cells were stimulated using immobilized αCD3 (0.5 μg ml^−1^) and αCD28 (0.5 μg ml^−1^). **b**, **c** Actinomycin D was added 18 h post stimulation and lysates were collected at indicated time points post-actinomycin D treatment. RNA was isolated post-stimulation and cDNA was synthesized for qRT PCR. All data are normalized to *18* *S* rRNA. Error bars indicate SEM; paired *t* test. **b**, **c** Data are representative of three to four independent experiments

We sought to determine the molecular mechanism by which IL-1R mediated signaling licenses effector functions of CD4 T cells. We found significantly reduced expression of T cell cytokine transcripts when IL-1R signaling was abrogated during reactivation of CD4 T cells (Fig. 6a). The IL-1R signaling pathway is analogous to TLR signaling as they both rely on homotypic interactions between TIR domain-containing proteins for signal transduction that culminate in activation of NF-κB and MAPK cascade^41^. TLR signaling is known to impose post-transcriptional control on innate cytokine transcripts such as *Tnfa*, *Ccl10* and *Il6* via the p38 MAPK pathway^42^. We hypothesized that MyD88 dependent signaling (downstream of IL-1 and IL-18 receptors) in CD4 T cells might regulate the post-transcriptional stability of effector cytokines, since pre-committed CD4 T cells are likely to induce transcription of effector cytokines soon after TCR activation and co-stimulation. Consistent with the idea that IL-1R signaling could be influencing the stability of cytokine transcripts, we found that IL-1β-mediated enhancement of cytokine transcripts was p38-dependent, as transcriptional expression of Th17 cytokines and *Il13* were reduced in the presence of a p38 MAP kinase inhibitor (Supplementary Fig. 7a). We further examined the sequence of signature effector cytokine mRNAs and found that those of *Il17a* and *Il13* contain conserved AU-rich motifs in their 3′ un-translated regions (Supplementary Fig. 7b, c). The presence of these motifs implies their susceptibility to RNA binding proteins^43^ which can destabilize these inflammatory transcripts, as also reported by other groups^44–47^. However, innate cues that can stabilize cytokine transcripts are not well characterized. This prompts the hypothesis that post-transcriptional stabilization is a function likely to be conserved between TLR and IL-1R signaling. Consistent with our hypothesis, abrogation of IL-1R signaling during reactivation led to rapid decay of *Il17a*, *Il17f*, *Il22*, *Il13*, and *Il5* transcripts (Fig. 6b). Conversely, presence of recombinant IL-1β during CD4 T cell reactivation (via TCR and co-stimulation) rescued destabilized cytokine transcripts (Fig. 6c). We did not observe enhanced transcriptional decay of *Ifng* in the absence of IL-1R signaling (Fig. 6b). This is in agreement with earlier reports suggesting a more complex epigenetically regulated mechanism of *Ifng* production^48–50^. Since other cell types such as macrophages, NK cells and CD8 T cells also produce IFNγ to exert their effector function, it is possible that the regulatory mechanisms of IFNγ production evolved distinctly from other T cell effector cytokines such as IL-17A and IL-13. These data establish that IL-1R signaling in T cells licenses cytokine production by providing post-transcriptional stability to effector cytokine transcripts. It is, however, possible that other additional mechanisms, such as IL-1-dependent de novo transcription and post-translational regulation, exist that need further investigation.

### Primed *Il1r*^*−/−*^ CD4 T cells show impaired cytokine production

**Fig. 7:**
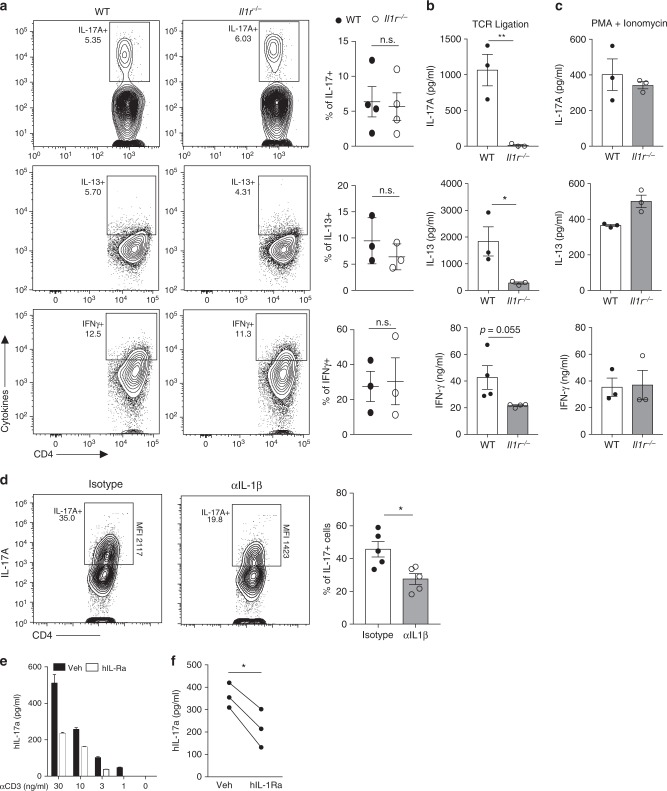
IL-1R signaling is critical for cytokine production by primed CD4 T cells in mice and in humans. **a**–**c** WT and *Il1r*^*−/−*^ mice were cohoused for at least 3 weeks. **a** Intracellular staining following activation of T_em_ with PMA + Ionomycin. **b** Secreted cytokines were measured 24 h following activation of splenic T_em_ with CD11c + DCs in the presence of soluble αCD3 (30 ng ml^−1^). **c** Secreted cytokines were measured 6 hours following activation of splenic T_em_ cells with PMA and Ionomycin. **d** In situ IL-17A production by CD3 + CD4 + CD44^hi^ T cells in LPLs from WT mice treated with αIL-1β or isotype control following in vivo T cell stimulation via intraperitoneal injection of αCD3 (20 μg). IL-17A MFI on CD4 + CD44hi cells is shown. Untreated mice serve as baselines for constitutive cytokine production by CD4 T cells. **e** IL-17A in the culture medium of human memory (CD4 + CD45RO + ) T cells reactivated with autologous MDDCs in the presence of αCD3 (OKT3, 30 ng ml^−1^) and hIL-1Ra (200 ng ml^−1^) for 24 h. Memory CD4 T cells and monocytes were isolated from peripheral blood of a healthy donor. Mean±SEM of technical duplicates are presented. **f** IL-17A in the culture medium of human memory T cells reactivated with autologous MDDCs from several donors in the presence of given concentrations of αCD3 (OKT3, 30 ng ml^−1^) and hIL-1Ra (200 ng ml^−1^) for 24 h. Each line represents an independent donor. Error bars indicate SEM; **b**–**d** unpaired *t* test; **f** paired *t* test

The majority of studies examining the role of IL-1R in CD4 T cell immunity use cytokine production as a measure of T cell response, making it challenging to ascertain the requirement of IL-1R signaling for lineage commitment versus effector function^18,21,22,51^. Our results showing the requirement of IL-1 for optimal cytokine production by successfully committed CD4 T cells dictated us to examine the status of Th1, Th2 and Th17 lineage cells in the spleen and the lamina propria of cohoused WT and *Il1r*^*−/−*^ mice. Consistent with previous reports^52–55^, we found no defect in the proportion of CD4 T cells committed to the Th17 lineage in the absence of IL-1R (Fig. 7a, Supplementary Figs. 1d, 8a) or MyD88 signaling (Supplementary Figs. 1d, 8d). This suggests that even though IL-1 is a critical player in CD4 T cell activation and Th17 differentiation following immunizations and infections^18,21,22,42^, other cues (IL-6, IL-23, etc.) might be able to drive Th17 lineage commitment of commensal-specific CD4 T cells in the absence of IL-1R signaling. We also found no defects in the proportion of cells that were committed to Th1 and Th2 lineage in the absence of IL-1R (Fig. 7a and Supplementary Fig. 1d). We took advantage of this IL-1R independent in vivo commitment of naturally arising effector CD4 T cells to ask if TCR-mediated stimulation is sufficient to drive cytokine production. We discovered that CD4 T cells that lacked IL-1R or MyD88 had impaired cytokine production when stimulated via their TCR (Fig. 7b, Supplementary Fig. 8b, e). This was not a result of impaired secretion since acute stimulation of the same CD4 T cells with phorbol 12-myristate 13-acetate (PMA) and Ionomycin could bypass the requirement of IL-1R signaling for IL-17A, IL-13 and IFNγ production (Fig. 7c and Supplementary Fig. 8c). These data establish that even though CD4 T cells can undergo effector lineage commitment in vivo in the absence of IL-1R signaling, they are unable to secrete effector cytokines without a functional IL-1R. PMA+ Ionomycin stimulation has long been used to reveal lineage commitment as well as effector function of CD4 T cells, as it provides necessary signals that can drive transcription of accessible loci^56^. In addition, PMA+ Ionomycin also induces activation of the p38 MAP kinase^57^, thus overriding the physiological dependence on IL-1 stimulus to drive cytokine production from already committed *Il1r*^*−/−*^ CD4 T cells. We demonstrate that lineage commitment does not ensure optimal cytokine production upon TCR activation since IL-1 remains a critical innate cue for effector function despite successful differentiation. These data provide critical in vivo genetic evidence for the physiological importance of IL-1R signaling in regulating effector function of pre-committed memory CD4 T cells.

### IL-1β is critical for IL-17A production in mice and humans

IL-1 has been linked to several auto-inflammatory disorders primarily because of its role in promoting Th17 responses. Naturally arising Th17 cells that are likely to cause auto-inflammation are primed against commensal microbiota and predominantly reside in the small intestine^15,58^. In contrast to CD8 T cells, the tools and experimental techniques for measuring direct effector function of memory CD4 T cells in vivo are limited^59–61^. It has been previously reported that simply ligating the TCR through injection of αCD3 leads to robust production of IL-17 by T cells in the small intestine^58^. Therefore, we adopted this approach to test if in vivo IL-17A production by pre-committed Th17 cells in the lamina propria is dependent on IL-1β. In order to investigate in situ Th17 effector function, CD4 T cells, isolated from the intestinal lamina propria of mice treated with αCD3, were cultured in the presence of brefeldin A without any further stimulation. In agreement with previous studies, we observed robust IL-17A production by T cells upon acute αCD3 treatment (Fig. 7d and Supplementary Fig. 1e). However, neutralization of IL-1β considerably compromised the IL-17A production by pre-committed CD4 T cells in vivo (Fig. 7d and Supplementary Fig. 1e). Interestingly, in vivo IL-17A production was compromised at the level of each cell, consistent with the role of IL-1R signaling in stabilizing IL-17A transcripts (Fig. 7d). Next, we extended our studies to circulating human Th17 cells. The hIL-1R antagonist, Anakinra, is a widely used drug for auto-immune ailments^62^. It has been shown that addition of exogenous IL-1β to primed human Th17 cells promotes IL-17A production^63,64^. Our mouse T cell data points to the possibility that Anakinra might also be abrogating Th17 effector function in addition to suppressing the other pathogenic effects of IL-1α or IL-1β. Indeed, we found that activation of circulating human memory CD4 T cells with autologous monocyte derived DCs in the presence of hIL-1R antagonist resulted in significantly diminished hIL-17A production over a wide range of avidity of DC-T cell interactions (Fig. 7e) and with multiple donors (Fig. 7f) suggesting a conserved role for IL-1R signaling in regulating memory Th17 effector function in both mice and humans.

## Discussion

T cells are exposed to a diverse array of innate cytokines throughout their lives that determines the quality of their response^2,6^. Our studies reveal that two distinct sets of innate cytokines control priming versus reactivation of CD4 T cells. It is well established that IL-12, IL-4 and IL-6 drive the differentiation program for Th1, Th2 and Th17 priming, respectively^6^. Here we show that the IL-1 family of cytokines plays a critical role during the reactivation of previously primed effector and memory CD4 T cells. This is true irrespective of whether the CD4 T cells are newly generated effector cells, circulating effector memory cells or tissue resident memory cells suggesting a conserved role for T cell-intrinsic MyD88 in regulating effector response. Interestingly, we found that while polyclonal Th1 cells do not rely on IL-18R signaling for IFNγ production, *L. monocytogenes*-specific IFNγ production is largely dependent on IL-18R signaling. This suggests that the role of each IL-1 family member could be dictated by microenvironment cues and detailed analysis is needed to assess the necessity of IL-1R, IL-18R and IL-33R by each CD4 T cell lineage following diverse immune challenges.

The IL-1 family of cytokines has an established role in synergizing with priming cytokines to promote clonal expansion and differentiation^6,25,27,65–67^. Previous studies^21,22,51,68^, including our own^18^, that used mice or T cells deficient for IL-1R, IL-18R or MyD88 cannot distinguish the role of the IL-1 family of cytokines in priming versus effector function since the readouts are always cytokine production. Here we show that, at steady state, CD4 T cells are able to commit to Th1, Th2 and Th17 lineages in the absence of IL-1R signaling. This is consistent with other studies reporting normal Th17 differentiation in MyD88- or IL-1R-deficient mice^52–55^. However, these committed CD4 T cells were unable to effectively secrete their cytokines upon TCR ligation. While IL-1 certainly contributes to the process of differentiation, our data reveal its absolute necessity for CD4 T cell effector function. Notably, the necessity of IL-1R signaling for effector cytokine production was apparent upon cognate TCR re-activation while treatment with PMA and Ionomycin masked the physiological requirement of IL-1 for effector CD4 T cell response. Our results underscore the importance of employing physiological methods of stimulation to deduce true biological requirements for functional immunity. By using in vivo primed CD4 T cells from WT mice and blocking IL-1R and IL-18R signaling only during reactivation, we have teased out the importance of both IL-1 and IL-18 in regulating effector function of primed CD4 T cells.

It is important to note here that presence of IL-1 does not bypass the requirement of TCR engagement or co-stimulation, but provides an additional layer of regulation for CD4 T cell effector function. This suggests that immunopathologies, such as Cryopyrin-associated periodic syndromes (CAPS), that lead to exaggerated levels of IL-1β are unlikely to cause aberrant production of effector cytokines by memory CD4 T cells, since TCR ligation and co-stimulation are necessary to elicit memory CD4 T cell functions. Furthermore, presence of the PRR ligands during CD4 T cell reactivation does not eliminate the requirement of IL-1R signaling for optimal functioning of CD4 T cells. When OVA-specific or pathogen-specific CD4 T cells are reactivated by DCs in the presence of endotoxin-free OVA or specific pathogen lysate (containing several PRR ligands), the central requirement for IL-1R signaling in CD4 T cells does not change. This suggests that IL-1R signaling controls cytokine production by effector and memory CD4 T cells not only under sterile reactivation conditions, but also during secondary pathogenic challenge. It will be interesting to test if the dependence on IL-1 for effector cytokine production is critical for keeping autoimmune cells in check and/or to curtail T cell-mediated immunopathology during pathogen-specific responses.

Our data also implies that DC-T cell cognate interaction results in biologically relevant quantities of active IL-1β production even during sterile reactivation. This is reconcilable with published work showing that memory CD4 T cell reactivation can occur independent of TLR stimulation^3^. It will be valuable to determine the underlying mechanisms that lead to IL-1β production in the absence of any apparent TLR or inflammasome ligands. Identification of the molecular players involved in IL-1β production resulting from DC-T cell interactions might uncover novel targets to treat autoimmunity as well as auto-inflammatory diseases.

One of the hallmarks of memory T cell reactivation is the rapid kinetics of response. Since effector T cells have already undergone differentiation, engagement of TCR during reactivation triggers transcription of poised loci, which results in respective cytokine production^69^. We have identified conserved AU-rich motifs in the 3’UTR of T cell effector cytokines that are likely to render these transcripts highly unstable^43^. Post-transcriptional stabilization by IL-1R signaling perhaps guarantees rapid kinetics of cytokine response while maintaining inflammatory cytokine transcripts under strict regulation. Such a phenomenon has been previously reported for TLR-dependent cytokines^42^. In addition to providing critical signals for productive effector function, dependence on IL-1R signaling might be particularly useful in rapidly triggering and terminating effector cytokine production, especially since these cytokines can have devastating effects on host tissues^70^. Post-transcriptional stabilization of inflammatory cytokine transcripts is perhaps a conserved function across cell types and receptor families that use MyD88 for signaling. Given the role of IL-1 family of cytokines in activating various innate lymphoid cells (ILCs), it will be interesting to see if ILCs also employ a similar mechanism for regulating their effector cytokine production in response to IL-1β and IL-18 from myeloid cells^32^.

In summary, our data provide compelling evidence for a broadly applicable “licensing” function of IL-1R signaling in enabling optimal effector function of pre-committed CD4 T cells. Even though Th1, Th2 and Th17 lineages exhibit differential requirements for priming cytokines, we demonstrate that all three lineages depend on IL-1R signaling to mount an effector response, illustrating convergent evolution across all T cell lineages. It is remarkable that the adaptive immune system, despite having sophisticated features to define specificity, co-opted this MyD88-dependent signaling pathway, thus highlighting the importance of evolutionarily conserved ancient pathways for host defense. In addition, this study sheds light on the complex cross-talk between the innate and adaptive immune compartments that extends beyond priming and differentiation and continues in a long-lasting manner to the reactivation phase of CD4 T immunity.

## Methods

### Antibodies for cell culture

Mouse CD3ε Biolegend 100331, Human CD3 Biolegend 317303, IL-1β (LEAF) Biolegend 503504, IL-1α (LEAF) Biolegend 503206, CD80 Tonbo Biosciences 40-0801-U100, CD86 Tonbo Biosciences 40-0862-U100, CD28 Tonbo Biosciences 40-0281-M001, CD62L biotin Biolegend 104404, CD11c biotin Biolegend 117303, anti-biotin microbeads Miltenyi Biotech 130-090-485

### Antibodies for flow cytometry

Mouse CD4 BV421 Biolegend 100443 (1:400), Mouse CD4 FITC Biolegend 100405 (1:400), CD44 AF700 Biolegend 103025 (1:400), CD44 pacific blue Biolegend 103020 (1:400), CD62L APC Tonbo Biosciences 20-0621-U100 (1:400), IL-6r Biotin Biolegend 115803 (1:100), IL-12rb1 PE RnD systems FAB1998P-025 (10ul/test), IL-4Ra PE Biolegend 144803 (1:100), IL-1R1 Biotin Biolegend 113503 (1:100), IL-18Ra APC Biolegend 132903 (5ul/test), IL-17A FITC Biolegend 506908 (1:300), IL-22 PE Biolegend 516404 (5ul/test), IFNg PE Biolegend 505807 (1:200), ICOS APC Biolegend 313509 (1:400), CD69 PE BD Biosciences 553237 (1:400), CD25 Biotin BD Biosiences (1:400), Zombie Yellow live dead stain Biolegend 423103 (1:500), Streptavidin BV421 Biolegend 405241 (1:400), CFSE Biolegend 423801 (1:1000), IL-17A purified Biolegend 505807 (1:1000), antiIL-17A biotin Biolegend 507002 (1:1000), Anti IL-22 purified Biolegend 516401 (1:250), Anti IL-22 biotin Biolegend 516407 (1:200), Anti IFNg purified Biolegend 505702 (1:1000), Anti IFNg biotin Biolegend 505804 (1:1000), Anti IL-17F Capture antibody eBioscience 14-7473-68 (1:200), Anti IL-17F Detection antibody eBioscience 13-7474-68 (1:250), Anti IL-13 purified eBioscience 14-7133-85 (1:125), Anti IL-13 biotin eBioscience 13-7135-85 (1:2000), Anti IL-4 purified Biolegend 504102 (1:200), Anti IL-4 biotin Biolegend 504202 (1:2000), Anti IL-5 purified Biolegend 504301 (1:500), Anti IL-5 biotin Biolegend 504402 (1:500)

### Mice

C57BL/6 wild-type control mice were obtained from the UT Southwestern Mouse Breeding Core Facility. *Il1*α^*−/−*^ and *Il1β*^*−/−*^ mice were provided to us by Fayyaz S. Sutterwala at Cedars Sinai. IL-1α^*−/−*^ mice were generated by Yochiro Iwakura, Tokyo University of Science. *Il1β*^*−/−*^ mice were generated by David Chaplin, UA at Birmingham. *Il1r*^*−/−*^ mice were purchased from The Jackson Laboratory. *Myd88*^*−/−*^ were generated by Shizou Akira, Osaka University and provided to us by Ruslan Medzhitov, Yale University. All mice were bred and housed in a specific pathogen-free facility at UT Southwestern Medical Center. For isolation of steady state CD4 memory T cells, mice were housed in conventional facility for 2-4 weeks before tissue isolation. All mouse experiments were done as per protocols approved by Institutional Animal Care and Use Committee (IACUC) at UT Southwestern Medical Center.

### Human donors

Healthy human donor blood was commercially obtained from Carter Blood Care, Dallas, TX. Age and Sex of the donors are masked and unknown to us.

### Bacteria

*C. rodentium* was cultured in nalidixic acid (30 μg ml^−1^) containing LB broth. *L. monocytogenes* was cultured in BHI.

### Isolation of CD44^hi^CD62L^**lo**^ cells from spleen and lymph nodes

Single cell suspension was obtained from spleen and peripheral lymph nodes. 4-8 mice were pooled for each experiment. CD4 T cells were isolated using mouse a CD4 T cells isolation kit (Biolegend) following manufacturer’s instructions. Cells were then labeled with a biotinylated CD62L antibody followed by anti-biotin microbeads (Miltenyi). CD62L^lo^ cells were isolated using negative selection by AutoMacs. Approximately 95% cells were CD4 + CD44 + CD62L^lo^.

### Isolation of splenic DCs

CD11C + cells were magnetically sorted from splenocytes using positive selection by AutoMacs. Approximately 95–99% cells were CD11c + MHCII+.

### Isolation of lamina propria lymphocytes

Small intestines were flushed with cold PBS and carefully cut longitudinally. 1-2 cm pieces of the tissue were digested twice with 2 mM EDTA buffer followed by 3 rounds of enzymatic digestion with Collagenase IV (100 μg ml^−1^, Sigma) and DNaseI (500 μg ml^−1^, Sigma). Cell suspension obtained after digestions was loaded on 40–70% percoll gradient as described before^18^.

### Memory T cell re-stimulation via TCR ligation in vitro

CD4 T cells were stimulated with either plate bound αCD3 (0.5 μg ml^−1^) + αCD28 (0.5 μg ml^−1^), or B cells (B cell:T cell = 2:1), or DCs (DC:T cell = 1:5) in the presence of 30 ng ml^−1^ soluble αCD3 for 24–48 h.

### Stimulation of CD4 T cells using PMA and ionomycin

CD4 T cells were treated with PMA (1 mg ml^−1^, Sigma) and ionomycin (100 nM, Sigma) for 4–6 h. For intracellular staining brefeldin A was also added.

### Re-stimulation of human memory T cells

PBMCs were isolated from healthy human donors using a Ficoll gradient. Memory CD4 T cells were isolated from healthy human PBMCs using a human memory CD4 T cell enrichment kit (Stem cell). For generation of MDDCs, autologous monocytes were isolated using a human Monocyte isolation kit (Stem Cells). Monocytes were then differentiated in the presence of recombinant GM-CSF (100 ng ml^−1^, RnD systems) and IL-4 (50 ng ml^−1^, RnD Systems) for 7 days.

### T cell re**-**stimulation in vivo

Approximately 6–8 weeks old mice were treated with 20 μg αCD3 or PBS i.p. αCD3 treated group was also treated with 50 μg of αIL-1β or isotype control on days −1, 0, and 1. Lamina propria cells were isolated 36 h after stimulation. All digestions were done in the presence of brefeldin A. Lamina propria lymphocytes were then cultured in the presence of brefeldin A for 4hrs immediately followed by surface and intracellular straining.

### Infections and immunizations

For the oral infection model, WT mice were treated with 200 μl 1% NaHCO_3_ followed by intragastric infection with 1 × 10^8^ CFU of *C. rodentium*. Mesenteric lymph nodes were excised 9–10 dpi. For the systemic infection model WT mice were infected with 20,000 CFU of *L. monocytogenes* i.p. Spleens were harvested 7 dpi. Infection dose was confirmed via retrospective counting. For antigen-specific reactivation, pathogen extracts were prepared via freeze thawing bacteria followed by total protein quantification. For subcutaneous immunization, 25 μg OVA (Invitrogen) and 2.5 μg LPS (Sigma) was emulsified in IFA and injected subcutaneously into footpad (hind limbs). Popliteal and inguinal lymph nodes were harvested 7 days post immunization.

### RNA stability quantification

Lymph node (5 × 10^6^ cells) cells were activated in the presence of soluble αCD3 (200 ng ml^−1^) for 18 h. Alternatively, CD4 T cells were isolated from the spleen and LNs and reactivated in vitro with immobilized αCD3 (0.5 μg ml^−1^) and αCD28 (0.5 μg ml^−1^). Cells were washed and plated at lower density (1 × 10^6^ cells ml^−1^) in the presence of actinomycin D (10 μg ml^−1^, Sigma). At given time points after addition of actinomycin D cells were washed with cold PBS and lysed with TriZol (Life Technologies) and frozen until further processing.

### Quantitative real-time PCR

RNA was isolated using Qiagen RNA extraction kit using manufacturer’s protocol. cDNA was synthesized using Random primers (Invitrogen) and MMLV reverse transcriptase (Invitrogen). The QuantStudio 7 Flex Real-Time PCR System (ThermoFisher Scientific) was used to measure SGBR green (ThermoFisher Scientific) incorporation. All data is normalized to 18 s rRNA. qPCR primers sequences are as follows: IL-1R1F: CAGGAGAAGTCGCAGGAAGT, IL-1R1 R: TGGAACAGAGCCAGTGTCAGIL17A F: TCCAGAAGGCCCTCAGACTA, IL17A R: AGCATCTTCTCGACCCTGAA, IFNg F: TGCCAAGTTTGAGGTCAACAACCCA, IFNg R: CCCACCCCGAATCAGCAGCG, IL17F F: ACCCGTGAAACAGCCATGGTCA, IL17F R: ACCGGTGGGGGTCTCGAGTG, IL22 F: CAATCAGCTCAGCTCCTGTCACAT, IL22 R: TCCCCAATCGCCTTGATCTCTCCA, IL13 F: ACAAGACCAGACTCCCCTGT, IL13 R: TCTGGGTCCTGTAGATGGCA, 18 S F: GTAACCCGTTGAACCCCATT, 18 S R: CCATCCAATCGGTAGTAGCG.

### Surface and intracellular staining and flow cytometry

Cells were stained with relevant antibodies for 30 min on ice and washed. For intracellular staining, Foxp3 staining buffer set (ebioscience) was used according to manufacturer’s protocol. The stained cells were analyzed with BD LSRII or Novocyte (ACEA biosciences). For cytokine receptor staining, control refers to fluorescence minus one control. Data were analyzed with FlowJo 10 Software.

### Quantification and statistical analysis

Based on previous and preliminary studies in our lab, we predicted that the reported samples sizes would be sufficient to ensure adequate power. Statistical analyses were performed in Prism (GraphPad) using unpaired or paired student’s *t* test as indicated in the figure legends. Data are presented as means±SEM. Significance was considered at *p < 0.05, **p < 0.005, ***p < 0.0005. n.s. = not significant.

### Data availability

The data generated during and/or analyzed during the current study are available from the corresponding author on reasonable request.

## Electronic supplementary material

Supplementary Information

Peer Review File
