# Supplementary material for: T cell-intrinsic IL-1R signaling licenses effector cytokine production by memory CD4 T cells
Source: Nat Commun. 2018 Aug 9;9:3185. doi: 10.1038/s41467-018-05489-7 (PMC6085393; doi:10.1038/s41467-018-05489-7)
Supplement: Supplementary file 1 — Supplementary Information [file 41467_2018_5489_MOESM1_ESM.pdf]

T cell-intrinsic IL-1R signaling licenses effector cytokine production by memory CD4 T cells

Jain et.al.

Supplementary Figure 1

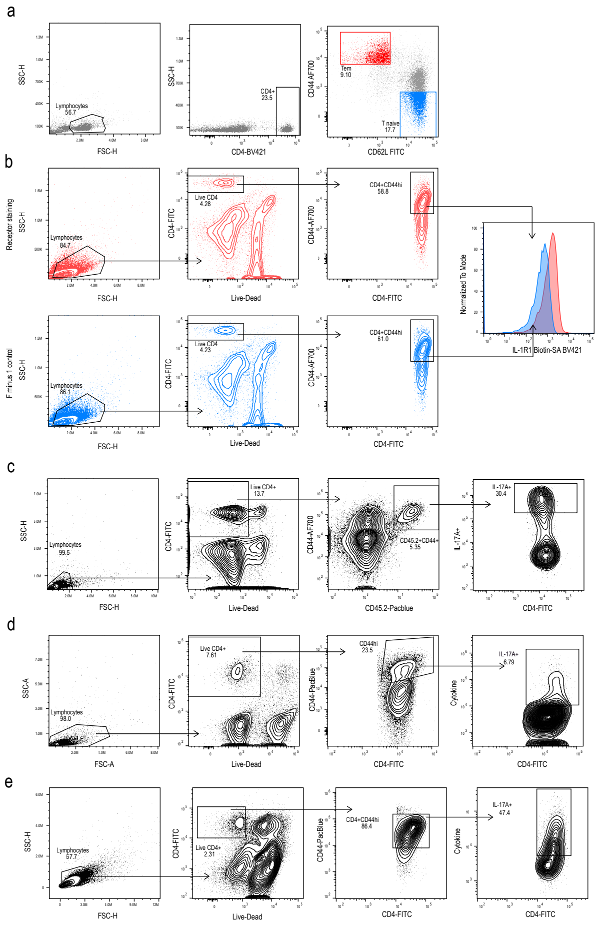

Supplementary Figure 1. Gating strategy used for the analysis of flow cytometry data (a) Gating strategy for Tem and Tnaive (b) Gating strategy to analyze receptor expression on SI-LP CD4 T cells. (c) Gating strategy to analyze cytokine production by donor OTII T cells. (d) Gating strategy to analyze cytokine production by endogenous CD4 T cells following antigen specific or polyclonal re-stimulation (e) Gating strategy to analyze cytokine production upon *in vivo* re-activation.

Supplementary Figure 2

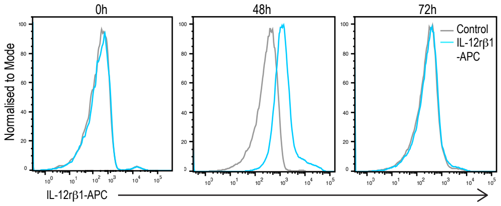

Supplementary Figure 2. Cytokine receptor expression on naïve and activated CD4 T cells.

Naïve CD4 T cells were stimulated with immobilized αCD3 (5 μg/ml) and αCD28 (5 μg/ml). Cells were stained at given time points following stimulation. Gated on Live CD4+ cells. Data are representative of two independent experiments.

Supplementary Figure 3

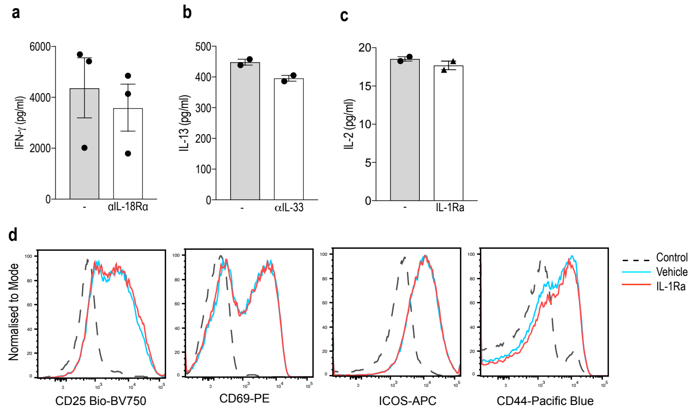

Supplementary Figure 3. Cytokine production and activation of status of CD4 T cells upon in vitro re-stimulation in the absence of IL-1 and related cytokine signaling.

(a and b) T_{em} were stimulated with WT CD11C+ splenic DCs and αCD3 (30 ng/ml) in the presence or absence of (a) αIL-18Ra (0.5 µg/ml) and (b) αIL-33 (100 ng/ml). (c) T_{em} were stimulated with WT CD11C+ splenic DCs and αCD3 (30 ng/ml) in the presence or absence of IL-1Ra for 48hrs (d) T_{em} cells were stimulated with CD11C+ splenic DCs and αCD3 (30 ng/ml) in the presence or absence of IL-1Ra for 36hrs followed by staining of respective T cell activation markers. Cells are gated on Live CD90.2+CD4+ events. Error bars indicate SEM; (d) Data are representative of three independent experiments.

Supplementary Figure 4

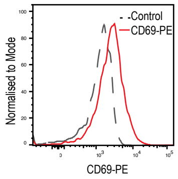

Supplementary Figure 4. CD69 staining on SI-LP CD4 T cells shows their tissue resident status.

Supplementary Figure 5

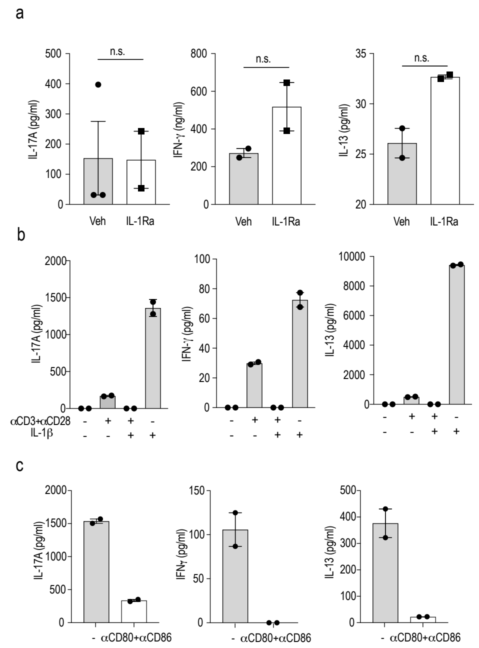

Supplementary Figure 5. IL-1R signaling does not bypass the requirement of co-stimulation

(a) T_{em} cells were stimulated with immobilized αCD3 (0.5 μg/ml) and αCD28 (0.5 μg/ml) in the presence or absence of IL-1Ra for 24 hrs. (b) T_{em} cells were stimulated with or without immobilized αCD3 (0.5 μg/ml) and αCD28 (0.5 μg/ml) and IL-1β (10 ng/ml) for 48 hrs. (c) T_{em} cells were co-cultured with WT splenic DCs and αCD3 (30ng/m) in the presence of CD80 (10 μg/ml) and CD86 (10 μg/ml) neutralizing antibodies. DCs were incubated with antibodies for 30min before addition of T cells. Error bars indicate SEM; (a) paired *t* test, n.s.= not significant.

Supplementary Figure 6

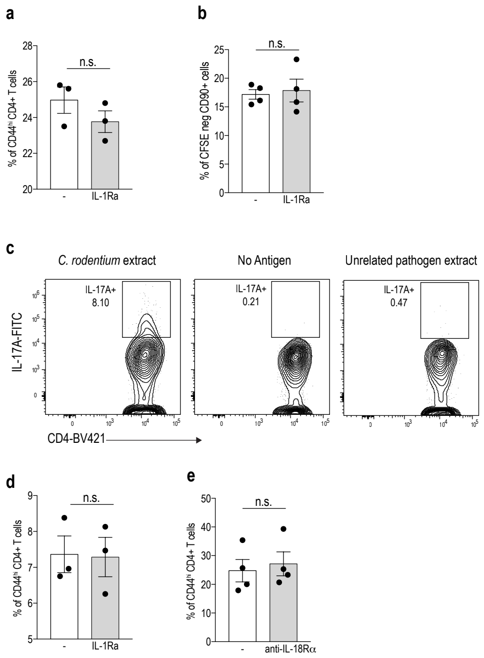

Supplementary Figure 6. Absence of bystander activation and no effect of IL-1Ra on activation and proliferation during antigen specific re-stimulation of T cells

(a) Frequency of CD44hiCD4+T cells upon OVA specific reactivation of CD4 T cells in the presence or absence of IL-1Ra. (b) Proportion of proliferating cells during OVA specific reactivation of CD4 T cells isolated from OVA immunized mice. (c) Frequency of IL-17A producing cells from mesenteric lymph nodes of *C. Rodentium* infected WT mice in the presence of given stimulation. Unrelated pathogen is *L. monocytogenes*. Frequency of CD44hiCD4+T cells upon (d) Cr and (e) Lm specific reactivation of CD4 T cells in the presence or absence of IL-1Ra and anti-IL-18Rα, respectively. (a, b, d and e) Error bars indicate SEM; n.s.= not significant; paired *t* test. (c) Data is representative of three independent experiments

Supplementary Figure 7

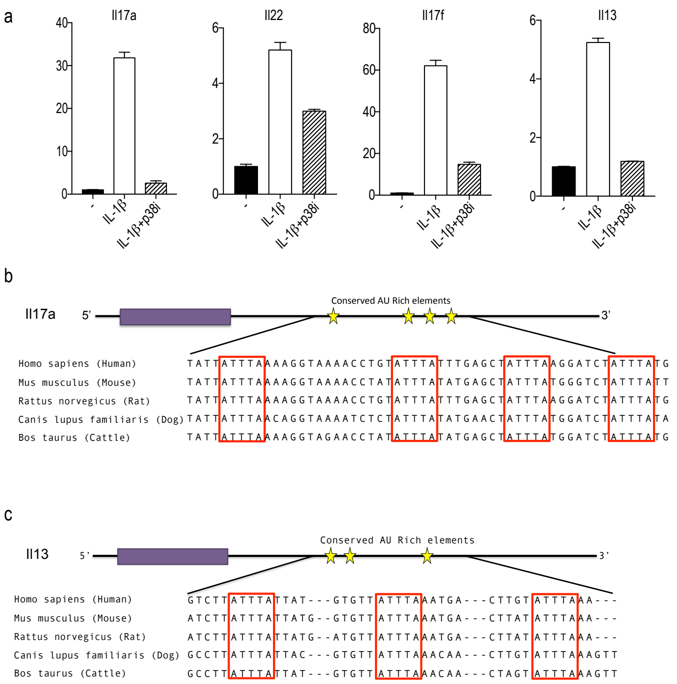

Supplementary Figure 7. Post-transcriptional regulation of CD4 T cell effector cytokines (a) Quantification of cytokine transcripts in activated purified CD4 T cells in the presence of IL-1β (20 ng/ml) and p38i (5 μM). (b and c) Multiple sequence alignment of 3'UTR of *Il17a* and *Il13* from various species. Error bars indicate SEM; Duplicates from one of the three independent experiments are presented.

Supplementary Figure 8

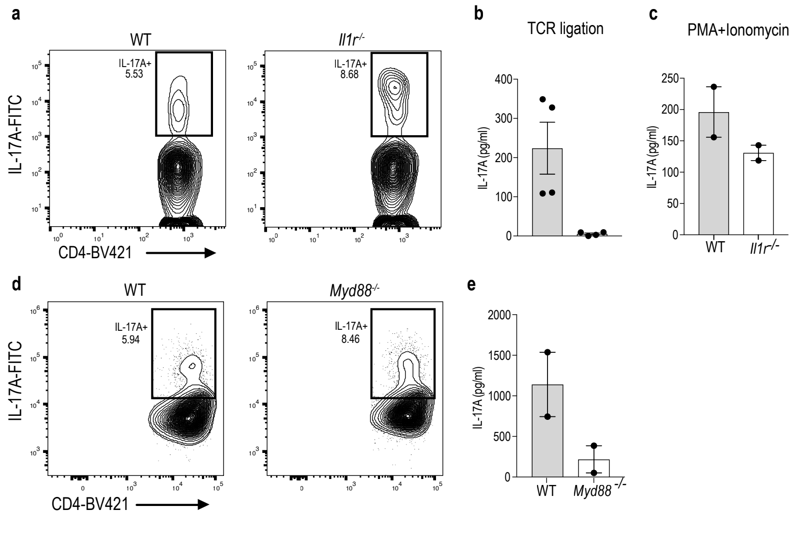

Supplementary Figure 8. Th17 lineage commitment and effector function of CD4 T cells in WT, *Il1r*$^{-/-}$ and *Myd88*$^{-/-}$ mice

(a) WT and *Il1r*$^{-/-}$ mice were cohoused for at least 3 weeks. (a) Proportion of IL-17A+ cells following activation with PMA + Ionomycin. (b and c) Secreted levels of cytokines following reactivation of splenic Tem with (b) PMA + Ionomycin (c) DCs in the presence of soluble αCD3. (d and e) WT and *Myd88*$^{-/-}$ mice were cohoused for at least 3 weeks. (d) Intracellular staining of cytokines following activation with PMA + Ionomycin (e) Secreted levels of cytokines following reactivation of splenic Tem with DCs in the presence of soluble αCD3. Error bars indicate SEM.
