# Supplementary material for: T cell-intrinsic IL-1R signaling licenses effector cytokine production by memory CD4 T cells
Source: Nat Commun. 2018 Aug 9;9:3185. doi: 10.1038/s41467-018-05489-7 (PMC6085393; doi:10.1038/s41467-018-05489-7)
Supplement: Supplementary file 2 — Peer Review File [file 41467_2018_5489_MOESM2_ESM.pdf]

Editorial Note: This manuscript has been previously reviewed at another journal that is not operating a transparent peer review scheme. This document only contains reviewer comments and rebuttal letters for versions considered at Nature Communications .

Reviewer #1 (Remarks to the Author):

The authors have basically addressed my concerns in the revised manuscript with new data and reasonable explanations.

Reviewer #2 (Remarks to the Author):

The authors have addressed my main concerns in establishing the T cell intrinsic nature of the requirement of IL-1R signaling. They also expanded on the models and added more data on tissue resident memory markers. The results shown are important to understand the different signals regulating memory T cells function, particularly in light of drugs which block IL-1R signaling.
